# Effect of COVID-19 on Stress and Biomarkers: An Exploratory Cross-Sectional Study

**DOI:** 10.7759/cureus.35702

**Published:** 2023-03-02

**Authors:** Amita Kumari, Afreen Begum H Itagi, Charushila A Rukadikar, Amudharaj D, Bijaya N Naik, Ayesha Juhi, Sunil Naik, Satish P Dipankar

**Affiliations:** 1 Physiology, All India Institute of Medical Sciences, Deoghar, Deoghar, IND; 2 Physiology, All India Institute of Medical Sciences, Mangalagiri, Mangalagiri, IND; 3 Physiology, All India Institute of Medical Sciences, Gorakhpur, Gorakhpur, IND; 4 Community and Family Medicine, All India Institute of Medical Sciences, Patna, Patna, IND

**Keywords:** immunity, anxiety, biomarkers, covid-19, stress

## Abstract

Background

Anxiety and stress in COVID-19 lead to continual pro-inflammatory cytokine activity resulting in excessive inflammation. Levels of different bio indices of COVID-19 may predict clinical outcomes and the severity of COVID-19 disease and may correlate with anxiety and stress levels.

Objectives

To measure the level of anxiety in COVID-19 patients using the coronavirus anxiety scale (CAS) as an assessment of psychological stress. To measure the levels of blood biomarkers and biochemical and hematological markers of inflammation in COVID-19. To record and measure the indices of short-term HRV in COVID-19 patients to assess their physiological and psychological stress levels. To determine the relationship between anxiety scores, levels of laboratory indices (blood biomarkers), and HRV parameters across mild, moderate and severe cases of COVID-19.

Material and method

A total of 300 COVID-19 patients aged between 18 and 55 years were included. A questionnaire-based CAS was used to assess anxiety levels. Short-term HRV was recorded to measure stress. Blood biomarkers: Biochemical and hemato-cytological markers of inflammation were measured. Statistical analyses were performed using the SPSS software version 20.0.

Results

Anxiety and stress increased with the severity of COVID-19. A positive correlation was detected between anxiety and serum ferritin, IL-6, MCV, and MCH levels, and a negative correlation between the corona anxiety score and RBC count. The increase in the severity of COVID-19 showed elevated levels of WBC count, neutrophil%, platelet count, neutrophil/lymphocyte ratio, serum ferritin, D-dimer, C-reactive protein, procalcitonin, interleukin-6, and lactate dehydrogenase, and decreased lymphocyte and monocyte percentages. The increase in the severity of COVID-19 decreased lymphocyte, monocyte, and eosinophil counts.

Conclusion

The Corona Anxiety Scale and heart rate variability can be used as complementary tools to index COVID-19-related anxiety and stress. An association exists between immune dysregulation and heart rate variability, which can be used to predict the inflammatory response and prognosis of COVID-19.

## Introduction

COVID-19: an outline

The coronavirus disease 2019 (COVID-19) pandemic may be stressful for people. The World Health Organization (WHO) declared the spread of coronavirus disease 2019 (COVID-19) as a pandemic on March 11, 2020 [1]. Everyone may have experienced increased stress during the pandemic. Fear and anxiety about a new disease and what could happen can be overwhelming and cause strong emotions in both adults and children. Public health actions, such as lockdowns, social distancing, and home quarantine, can make people feel isolated and lonely and can increase stress and anxiety [2]. The global mortality rate is about 3.4% (WHO, 2020), and observational studies indicate that pre-existing conditions such as obesity, cardiovascular disease, diabetes, chronic respiratory disease, hypertension, and cancer increase this rate [3]. Patients infected with COVID-19 can exhibit a wide range of clinical manifestations ranging from an asymptomatic state to severe disease with hypoxia and acute respiratory distress syndrome-type lung injury. Few patients experience hypoxemic respiratory failure and ground-glass opacification on chest imaging, while most patients experience only mild symptoms, including fever, cough, headache, anorexia, diarrhea, malaise, loss of smell, and taste sensation. All these factors lead to anxiety and stress situations among COVID-19 patients [4].

Stress in COVID-19

Any internal or external state that can activate the Hypothalamic-Pituitary Axis (HPA) can be defined as stress. Stress can be broadly divided into, but not limited to, psychological stress and physiological stress. Although both types of stress can ultimately result in the activation of the HPA axis and the Sympathetic Nervous System (SNS), they differ significantly in their mode, timing, and severity. Psychological stress due to anxiety during an infectious disease outbreak causes fear and worry about one’s health and the health of loved ones, financial situations or jobs, loss of support services, changes in sleep or eating patterns, difficulty in sleeping or concentrating, worsening of chronic health problems and mental health conditions, and increased use of tobacco, alcohol, and other substances. Physiological Stress results in a state of “allostasis,” in which the sympathetic nervous system is activated along the HPA axis. It is important to note that COVID-19 results in inflammation, activation of the sympathetic nervous system, and increased inflammation through complex and intricate connections with various components of the immune system.

Keeping in mind that the outbreak of COVID-19 may be stressful for people, Sherman A. Lee is an associate professor of psychology at Christopher Newport University in Virginia. He is actively researching COVID-19-associated anxiety and has developed the first medical screening tool for this condition, the coronavirus anxiety scale (CAS). The CAS is a brief mental health screener used to identify probable cases of dysfunctional anxiety associated with the COVID-19 crisis. CAS is physiologically based on dysfunctional anxiety symptoms associated with the COVID-19 crisis. The CAS is currently being translated into many different languages and is being used by over 50 teams of health professionals and researchers worldwide. [5]. Therefore, it is necessary to study COVID-19-induced psychological stress during this pandemic. Our purpose was to measure the level of stress during this time and characterize it according to location, gender, income, and other factors. Therefore, we planned to measure the anxiety and stress of COVID-19-positive patients and study the correlation between them.

COVID-19 and blood biomarkers (biochemical and hemato-cytological parameters of inflammation)

In recently published research articles, it was found that several laboratory indices may facilitate the assessment of COVID-19 severity, such as low lymphocyte count as well as serum levels of C-reactive protein (CRP), D-dimer, ferritin, cardiac troponin, and interleukin (IL-6), which may be used in risk stratification to predict severe and fatal COVID-19 patients [6,7]. Therefore, we planned to collect the patients’ hemato-cytological and biochemical parameters of COVID-19 inflammation. Hematological indices included mean corpuscular volume (MCV), mean corpuscular hemoglobin (MCH), mean corpuscular hemoglobin concentration (MCHC), mean platelet volume (MPV), neutrophil-to-lymphocyte ratio (NLR), procalcitonin (PCT), packed cell volume (PCV), platelet distribution width (PDW), red blood cell (RBC), red blood cell distribution width-coefficient of variation (RDW-CV), and white blood cell count (WBC). The biochemical indices of inflammation include C-reactive protein (CRP), D-dimer, serum ferritin, hemoglobin (Hb), interleukin-6 (IL-6), lactate dehydrogenase (LDH), which are routinely done in COVID-19 patients admitted in AIIMS Patna. These indices have been found to increase inflammatory states that are proportional to the severity of inflammation.

COVID-19 and heart rate variability (HRV)

HRV is the fluctuation in the length of heartbeat intervals. It represents the heart's ability to respond to various physiological and environmental stimuli [8]. The time and frequency domains of short-term HRV have been time-tested and established as tools for measuring parasympathetic and sympathetic nervous systems. In the present study, we were interested in observing the state of the sympathetic nervous system. The indices of short-term HRV, such as LF power and LF/HF ratio, reflect the state of the sympathetic nervous system, and indices such as RMSSD, NN50, pNN50, and HF powers indicate the state of the parasympathetic nervous system (PNS). Indices reflecting SNS are expected to increase and indices of PNS activity are expected to decrease with increasing levels of inflammatory indices in COVID-19 patients [9]. Impaired health and related stress are linked to the sympathovagal imbalance, which is reflected in HRV measurement [10]. In the present study, we correlated COVID-19-related stress, which was measured using the CAS score and heart rate variability (HRV), with biochemical, hematological, and inflammatory indices in three different severity groups (mild, moderate, and severe) of COVID-19 patients.

## Materials and methods

Study design

This study adopted an exploratory cross-sectional analytical design. The study was conducted for three months from December 2020 to February 2021

Study setting

The study was conducted at the All India Institute of Medical Sciences (AIIMS), Patna, which is the center of excellence in the state of Bihar and was designated as a dedicated COVID-19 care hospital in the Bihar state of India during the COVID-19 pandemic. This research was conducted with the approval of the Institutional Ethical Committee (vide letter no. AIIMS/Pat/IEC/2020/630).

Study participants

Inclusion Criteria

COVID-19 patients of both genders aged 18-55 years diagnosed with the positive nasopharyngeal swab reverse transcription polymerase chain reaction (RT-PCR) test for severe acute respiratory syndrome coronavirus 2 (SARS-CoV-2) were included. Patients admitted to AIIMS Patna Hospital for COVID-19 treatment during the COVID-19 pandemic were grouped as a mild group: RT-PCR test for SARS-CoV-2 positive but no evidence of breathlessness or hypoxia (normal saturation), moderate group: RT-PCR test for SARS-CoV-2 positive with the presence of hypoxia SpO_2_ < 94% (range 90%-94%) and respiratory rate more or equal to 24 breaths/minute, and severe group: RT-PCR test for SARS-CoV-2 positive with clinical signs of pneumonia, SpO_2_ < 90% respiratory rate > 30 breaths/minute. This grouping of mild, moderate and severe cases was done following the Clinical Management Protocol COVID-19 released by the Ministry of Health and Family Welfare, Government of India [11].

Exclusion Criteria

Seriously ill patients (comatose/unconscious/on the ventilator), alcoholics, psychiatric care with antipsychotics, and antiarrhythmic drugs, patients with underlying non-communicable diseases like diabetes, hypertension, cardiac disease, chronic lung disease, cerebrovascular disease, chronic kidney disease, immunosuppression and cancer, patients who did not consent to participate in the study.

Study sample size and sampling technique

Owing to the lack of published literature, an explorative cross-sectional design was adopted. Hence, the sample size was not calculated, but all eligible 300 COVID-19 patients admitted at AIIMS, Patna during the study period, and who satisfied the inclusion-exclusion criteria were included in the study. We adopted census enrollment, and participants were enrolled consecutively during the study period.

Study tools

We used a pre-tested semi-structured questionnaire for data collection. The remainder of this paper is organized as follows. (i) Participant’s details, including history: General information of the study participants, such as name, age, sex, address, occupation, and history of COVID-19 progress in case of patients were included in the study. (ii) Investigation details of study participant's SpO_2_, respiratory rate, body temperature, complete blood count (Hb, RBC, WBC, PCV, MCV, MCH, MCHC, and RDW-CV), inflammatory indices (neutrophil%, lymphocyte%, monocyte%, eosinophil%, basophil%, MPV, PDW, platelet count, NLR, serum ferritin, IL-6, LDH, CRP, D-dimer, and PCT) were collected from the patient's case sheet, and stress indices (SDNN, RMSSD, PNN50, LF, and HF) were recorded for 5 min with the Elite CorSense HRV device. (iii) Study instruments are as follows.

Pulse Oximeter

The device detects hypoxemia, which is one of the early signs of coronavirus infection. Hence, we used it to assess the oxygen levels (SpO_2_), respiratory rate, and prognosis of COVID-19 management. 

Infrared Thermometer

We used it to detect body temperature and fever. This thermometer checks human temperature by sensing the infrared energy radiated by the body from a distance.

Heart Rate Variability Using Elite CorSense HRV

The Elite CorSense HRV device is a sensor-finger-based device that connects to an Elite HRV app on a smartphone via Bluetooth. CorSense’s HRV sensor is one of the most accurate and easy ways to get this data consistently [12,13].

Corona Anxiety Scale (CAS)

Sherman CAS is a brief questionnaire that identifies dysfunctional anxiety induced by the COVID-19 pandemic [14]. The CAS is a 5-item mental health screener designed to efficiently and effectively aid healthcare professionals and researchers in identifying probable cases of dysfunctional anxiety associated with the COVID-19 crisis. Each item of the CAS is rated on a 5-point scale, from 0 (not at all) to 4 (nearly every day), based on experiences over the past two weeks. A CAS total score ≥ 9 indicates probable dysfunctional coronavirus-related anxiety [15].

Statistical analysis

The collected data were entered into a Microsoft Excel file and analyzed using SPSS version 20.0. Continuous variables were expressed as mean and standard deviation (SD) depending on the normality distribution. Categorical variables were expressed as proportions. The stress (HRV) index levels were expressed as absolute values with 95% confidence intervals. A one-way ANOVA/Friedman test was performed to assess the levels of various stress indices in the three severity categories of COVID-19 patients. We performed multiple comparisons among groups using the Scheffe Post Hoc test after ANOVA for significant variables. For non-normally distributed variables, multiple Kruskal-Wallis tests with Bonferroni corrections were performed. Post-hoc analysis was performed using the Mann- Whitney test for multiple binary comparisons. To determine the correlation between stress indices and hematological indices, the Pearson correlation test was performed. A two-sided p-value of 0.05 or less was considered statistically significant and a p-value of 0.005 or less was considered statistically highly significant.

## Results

A total of 300 COVID-19 patients were included in the study. Nearly two-thirds of the patients were male, and the mean (SD) age was 44.5 (14.8) years. The median age was significantly higher among the patients with severe COVID-19. No significant differences in sex distribution were observed between the severity groups of COVID-19 (Table 1).

**Table 1:** Demographic and baseline characteristics of patients with COVID-19 A total of 300 COVID-19 patients (213 males and 87 females) were included in the study. Nearly two-thirds of the patients were male, and the mean (SD) age was 44.5 (14.8) years. The mean age was significantly higher among the patients with severe COVID-19. Significant differences in sex distribution were observed between the severity groups of COVID-19 (P<0.05).

		Mild	Moderate	Severe	P-value (Overall)
	Male (n=213)	112 (52.6%)	26 (12.27%)	75 (35.2%)	0.052
Gender	Female (n=87)	59 (67.8%)	8 (9.2%)	20 (23.0%)	
Age		41 (25-52)	48 (32.8-58.3)	52 (45-60)	<0.001*

Analysis was carried out on patients with data on CAS, HRV indices as well as hematological and inflammatory indices, values are depicted in Table 2.

**Table 2:** Association between CAS, HRV, and biomarkers NB: Normally distributed across COVID-19 categories; $Not normally distributed across COVID-19 categories; *Statistically significant:  p < 0.05 [One way ANOVA, Kruskal Wallis, Scheffe post hoc test]; **Statistically highly significant: p < 0.005 (Multiple Mann-Whitney tests and after considering Bonferroni correction) The statistical test used: One-Way ANOVA [normally distributed variables]; Kruskal –Wallis test [not normally distributed variables ($)], statistically significant level = p-value <0.05. The Scheffe post hoc test was performed for statistically significant normally distributed variables. Statistical significance was set at p-value < 0.05. For statistically significant non-normally distributed variables, post hoc analysis was performed using the Mann-Whitney test for multiple binary comparisons. After Bonferroni correction, a p-value of < 0.005 was considered statistically highly significant.

Association of Corona Anxiety Score with the severity of COVID-19							
Indices/Parameter	Mild Disease (mean ± SD)	Moderate disease (mean ± SD)	Severe disease (mean ± SD)	P-value	Post-hoc analysis P-value		
Indices				Overall	Mild vs Moderate	Mild Vs Severe	Moderate vs Severe
Corona Anxiety score (CAS)	14.485 ± 3.6338	15.265 ± 2.7777	16.968 ± 2.7032	<0.001**	0.449	0.001**	0.035*
Association of Autonomic Stress Parameters (HRV) with the severity of COVID-19							
LF (ms^2^)	92.84 (40.65-1672.10)	512.51 (11.92-7637.00)	16.26 (2.95-121.70)	<0.001**	0.106	<0.001**	<0.001**
HF (ms^2^)	73.44 (23.65-1220.73)	504.75 (6.07-6687.28)	31.52 (2.95-55.62)	<0.001**	0.218	<0.001**	<0.001**
LF/HF Ratio	1.26 (0.84-1.37)	1.14 (1.02-3.82)	1.57 (0.84-2.19)	0.319			
Association of Hematological parameters with the severity of COVID-19							
HB	12.054 ± 2.3764	11.144 ± 2.5224	11.466 ± 2.3318	0.43			
RBC	4.3496 ± 1.56048	3.9838 ± 0.88129	4.0333 ± 0.87198	0.098			
PCV	36.782 ± 6.7990	33.900 ±7.2896	34.961 ± 6.8010	0.025*	0.083	0.118	0.741
MCV	88.423 ± 7.4549	85.350 ± 6.1545	88.741 ± 7.8722	0.062			
MCH	28.864 ± 2.8977	28.035 ± 2.5855	28.773 ± 3.0594	0.317			
MCHC	32.621 ± 1.4120	32.818 ± 1.2629	32.557 ± 1.4197	0.647			
RDW-CV	14.588 ± 2.2567	14.141 ± 1.7840	14.699 ± 2.0059	0.421			
WBC	7.09 (5.77-9.93)	8.29 (6.13-11.79)	11.8066 ± 5.37708	<0.001**	0.158	<0.001**	0.008**
Association of immunologic and biomarkers with the severity of COVID-19							
Indices/Parameter	Mild disease (mean ± SD)	Moderate disease (mean ± SD)	Severe disease (mean ± SD)	p-value	Post-hoc analysis p-value		
Biomarkers				overall	Mild vs Moderate	Mid vs Severe	Moderate vs Severe
Neutrophil%	72.260 ± 15.6012	80.347 ± 10.9897	85.699 ± 10.4214	<0.001**	0.008**	<0.001**	0.149
Lymphocyte%	21.90 (10.90-29.8)	12.15 (7.52-21.62)	8.90 (5.6-16.7)	<0.001**	0.001**	<0.001**	0.52
Monocyte%	3.60 (2.50-5.30)	3.200 ± 1.1672	2.2 (1.4-3.1)	<0.001**	0.071	<0.001**	0.001**
Eosinophil%	0.50 (0.00-2.10)	0.20 (0.00-1.10)	0.00 (0.00-0.300)	<0.001**	0.206	<0.001**	0.006**
Basophil%	0.20 (0.10-0.30)	0.10 (0.10-0.30)	0.20 (0.10-0.30)	0.319			
MPV	13.364 ± 1.7120	13.300 ± 1.2933	13.225 ± 1.8980	0.821			
PDW	17.863 ± 3.4254	17.535 ± 3.4566	17.736 ± 3.2366	0.753			
Platelet count	177.409 ± 71.1793	181.118 ± 87.0036	207.979 ± 90.7152	0.011*	0.970	0.012*	0.243
NLR	3.41 (2.15-7.82)	6.37 (3.46-11.97)	9.82 (4.77-16.39)	<0.001**	0.001**	<0.001**	0.027*
Serum Ferritin	391.1777 ± 432.40058	563.4529 ± 501.22273	1021.3109 ± 576.30270	<0.001**	0.175	<0.001**	<0.001**
D-dimer	0.58 (0.34-1.16)	0.68 (0.48-1.40)	1.79 (0.66-11.0)	<0.001**	0.180	<0.001**	0.003**
CRP	22.46 (3.55-56.98)	74.77 (40.63-115.07)	96 (47.05-201.12)	<0.001**	<0.001**	<0.001**	0.052
IL-6	9.30 (3.9-15.0)	20.05 (5.40-41.75)	46.4 (10.0-205.0)	<0.001**	0.006**	<0.001**	0.010**
LDH	496.79 (347.10-788.20)	712.49 (529.56-845.11)	868.17 (649.12-1236.50)	<0.001**	0.002**	<0.001**	0.001**
PCT	0.09 (0.06-0.13)	0.12 (0.07-0.47)	9.82 (4.77-16.39)	<0.001*	0.014*	<0.001**	0.004**

Association of corona anxiety score with the severity of COVID-19

The difference in CAS scores was significant among the severity groups of COVID-19 patients. It has been found to increase with the severity of COVID-19. CAS score was significantly higher in the severe group compared to the mild group [(16.968 ± 2.7032) vs (14.485 ± 3.6338)] and in the severe group compared to the moderate group [(16.968 ± 2.7032) vs (15.26 ± 2.777)]. The CAS scores did not differ significantly between the moderate and mild COVID-19 groups.

Association of autonomic stress parameters with the severity of COVID-19

LF (ms2), HF (ms2), and HF (Hz) were significantly different among the severity groups of COVID-19 patients. LF (ms2) was significantly lower in the severe group than in the mild group [16.26 (2.95-121.7) vs 92.84 (40.65-1672.1]) and in the severe group than in the moderate group. [16.26 (2.95-121.7) vs 512.5 (11.92-7637.00)]. HF (ms2) was significantly lower in the severe group than in the mild group [31.52 (2.95-55.62) vs 73.44 (23.65-1220.73]) and in the severe group than in the moderate group. [31.52 (2.95-55.62) vs 504.75 (6.07-6687.28)]. The HF (Hz) was found to be significantly different among the severity groups of COVID-19 patients. HF (Hz) was significantly lower in the severe group compared to the mild group [0.17 (0.16-0.48) vs (0.3235 ± 0.1075)]. LF (Hz) was not significantly different among the severity group of COVID-19 patients.

Association of hematological parameters with the severity of COVID-19

In our study, levels of Hb, RBC, PCV, MCV, MCH, MCHC, MPV, lymphocyte%, monocyte%, eosinophil%, basophil%, and PDW were lower in patients with moderate and severe COVID-19 than in patients with mild COVID-19 where these values are closer to the normal level. Values of MCV, MCH, MCHC, MPV, basophil%, and PDW were statistically not significant; therefore, weak correlation was found. In contrast, WBC count, neutrophil%, platelet count, and NLR were increased. WBC count was significantly higher in the severe group compared to the mild group [(11.8066 ± 5.37708) vs 7.09 (5.77-9.93)] and in the severe group compared to the moderate group [(11.8066 ± 5.37708) vs 8.29 (6.13-11.79)]. The WBC count difference was not significant between the moderate and mild COVID-19 groups. Neutrophil% was significantly higher in the severe group than in the mild group [(85.699 ± 10.4214) vs. (72.260 ± 15.6012)] and the moderate group than in the mild group [(80.347 ± 10.9897) vs (72.260 ± 15.6012)]. Neutrophil% was not significantly different when comparing moderate to severe COVID-19. Lymphocyte% was significantly lower in the severe group compared to the mild group [8.90 (5.6 -16.7) vs 21.90 (10.90-29.8)] and in the moderate group compared to the mild group [(12.15 (7.52-21.62) vs 21.90 (10.90-29.8)]. The lymphocyte% was not significantly different between patients with moderate to severe COVID-19. Monocyte% was significantly lower in the severe group than in the mild group [2.2 (1.4-3.1) vs. 3.60 (2.50-5.30)] and in the severe group than in the moderate group [(2.2 (1.4-3.1) vs. (3.200 ± 1.1672)]. Monocyte% was not significantly different when comparing moderate to mild COVID-19. Eosinophil% was significantly lower in the severe group than in the mild group [0.00 (0.00-0.300) vs. 0.50 (0.00-2.10]) and in the severe group than in the moderate group [(0.00 (0.00-0.300) vs. 0.20 (0.00-1.10]). Eosinophil% was not significantly different between patients with moderate-to-mild COVID-19. Platelet count was significantly higher in the severe group than in the mild group [(207.979 ± 90.7152) vs. 177.409 ± 71.1793)]. The NLR was significantly higher in the severe group than in the mild group [9.82 (4.77-16.39) vs. 3.41 (2.15-7.82]) and the moderate group than in the mild group [6.37 (3.46-11.97) vs. 3.41 (2.15-7.82]). The NLR was not significantly different when comparing moderate to severe COVID-19.

Association of immunological indices with the severity of COVID-19

D-dimer, CRP, IL-6, LDH, and procalcitonin levels increased from mild to severe COVID-19. The level of D-dimer was significantly higher in the severe group compared to the mild group [1.79 (0.66-11.0) vs 0.58 (0.34-1.16)] and in a severe group compared to the moderate group [1.79 (0.66-11.0) vs 0.68 (0.48-1.40)]. D-dimer levels were not significantly different between patients with moderate-to-mild COVID-19. CRP levels were significantly higher in the moderate group than in the mild group [74.77 (40.63-115.07) vs 22.46 (3.55-56.98)]. Levels of IL-6 were significantly higher in the severe group compared to the moderate group [46.4 (10.0 -205.0) vs 20.05 (5.40-41.75)] and in the moderate group compared to the mild group [20.05 (5.40-41.75) vs 9.30 (3.9-15.0)]. IL-6 levels were significantly different between the severe and mild COVID-19 groups. LDH levels were significantly higher in the severe group than in the moderate group [868.17 (649.12-1236.50) vs. 712.49 (529.56-845.11]) and in the moderate group than in the mild group [712.49 (529.56-845.11) vs. 496.79 (347.10-788.20]). LDH levels were significantly different between the patients with severe and mild COVID-19. Levels of PCT were significantly higher in the severe group compared to the moderate group [9.82 (4.77-16.39) vs 0.12 (0.07-0.47)], compared to the mild group [9.82 (4.77-16.39) vs 0.09 (0.06-0.13)] and in a moderate group compared to mild group [0.12 (0.07-0.47) vs 0.09 (0.06-0.13)].

## Discussion

In our study, anxiety scores were measured using the CAS. Our results revealed a weak correlation between anxiety and serum ferritin, IL-6, MCV, and MCH and a negative correlation between CAS score and RBC count. According to the study of Mazza et al. immune response and systemic inflammation based on peripheral lymphocyte%, neutrophil%, and platelet counts, are positively associated with scores of anxiety and depression [16].

Sympathetic activation and parasympathetic inhibition in COVID-19

Studies have shown widespread inflammation in COVID-19, and there is evidence in the scientific literature that the sympathetic nervous system is stimulated by inflammation [16]. The initial response to sympathetic activation promotes inflammation. There is also vagal inhibition in cases of inflammation, which has been documented in the literature [17]. There is also evidence that an activated sympathetic nervous system provokes more inflammation, at least in the initial phases [18]. Similar results were observed in the present study. In Table 3, reduced HRV predicted the severity in the severe group. The LF band which represents sympathetic activity was significantly higher in the severe group compared to the mild group [16.26 (2.95-121.70) vs 92.84 (40.65-1672.10)] and in the severe group compared to the moderate group [16.26 (2.95-121.70) vs 512.51 (11.92-7637.00)]. Therefore, LF P-value was found highly significant in the moderate and severe groups.

**Table 3:** Correlation between biomarkers and HRV (physiological stress) indices The correlation between the inflammatory indices and stress indices was weak or very weak (Pearson correlation coefficient [r] < 0.3). The statistical significance and directionality of the correlation between the inflammatory indices and stress indices are provided in the table. NB: Statistical test used: Pearson correlation test, the figure presented in the cells: Pearson correlation coefficient. *P < 0.05, **P < 0.005). LF: Low Frequency, HF: High Frequency, RMSSD: Root Mean Square Successive Difference, PNN50: Proportion of R-R intervals differing from their directly adjacent R-R intervals > 50ms, SDNN: Standard Deviation of Normal-Normal RR intervals, HF/LF ratio: ratio used to measure sympathovagal balance.

Biomarkers (hematologic, biochemical & inflammatory)	Coronavirus anxiety score	HRV (stress) indices					
		Sympathetic indices	Parasympathetic indices			Sympathovagal balance	
		LF (ms^2^)	HF (ms^2^)	RMSSD (ms)	PNN50 (%)	SDNN (ms)	HF/LF ratio
Ferritin	0.26**	-0.01	-0.03	0.14*	-0.08	-0.07	-0.14*
D-dimer	0.09	-0.03	0.09	-0.05	-0.08	-0.04	-0.01
C-RP	0.05	0.01	0.13*	0.08	-0.06	-0.02	-0.10
IL-6	0.15**	0.36**	0.17**	0.19**	-0.11	-0.08	-0.16**
LDH	0.15**	-0.04	-0.02	0.03	-0.09	-0.07	-0.16**
Hb%	-0.03	-0.16**	-0.08	-0.11	-0.06	-0.05	0.04
WBC	0.05	-0.03	-0.02	0.05	-0.07	-0.02	0.06
Platelet	0.02	-0.09	-0.09	.052	-0.02	-0.03	-0.01
RBC	-0.14*	-0.11	-0.06	-0.04	-0.05	0.09	-0.02
PCV	-0.05	-0.17**	-0.09	-0.13*	-0.07	-0.06	0.06
MCV	0.12*	0.07	0.04	0.06	-0.02	-0.05	0.09
MCH	0.12*	0.05	0.03	-0.04	0.01	-0.004	-0.01
MCHC	0.08	-0.02	0.00	0.01	0.06	0.06	-0.16**
RDW-CV	-0.04	0.00	-0.02	-0.03	-0.06	0.01	0.05
MPV	0.00	-0.03	-0.01	-0.04	-0.05	0.01	0.13*
PDW	0.00	-0.00	0.07	0.03	-0.03	-0.04	-0.00
Neutrophil%	0.16**	0.05	0.07	0.10	-0.06	0.09	-0.18**
Lymphocyte%	-0.18**	-0.04	-0.07	-0.10	0.05	-0.10	0.19**
Monocyte%	-0.19**	-0.04	-0.04	-0.07	0.25**	-0.03	0.13*
Eosinophil%	-0.00	-0.03	-0.04	-0.01	0.12*	-0.01	0.09
Basophil%	-0.02	-0.06	-0.05	0.02	-0.04	-0.11	0.01
PCT	0.08	0.02	-0.00	0.01	-0.09	-0.09	0.12*
NLR	0.17**	0.00	0.03	0.00	-0.09	-0.03	-0.15**

The HF band which represents parasympathetic activity was significantly lower in the severe group compared to the mild group [773.44 (23.665-1220.73) vs 31.52 (2.95-55.62)] and in the severe group compared to the moderate group [504.75 (6.07-6687.28) vs 31.52 (2.95-55.62)]. Therefore, HF P-value was found highly significant in the moderate and severe groups. In our study, Lower HF power is correlated with stress and anxiety. HF is highly correlated with the pNN50 and RMSSD time-domain measure. It has a positive correlation between mild to severe groups of COVID-19. The modulation of vagal tone helps maintain the dynamic autonomic regulation important for cardiovascular health. Deficient vagal inhibition is implicated in increased morbidity.

HRV and anxiety in COVID-19

Over the past few decades, research has shown a relationship between low HRV and worsening depression or anxiety. Low HRV is associated with an increased risk of death and cardiovascular diseases. An HRV measured during their natural breathing pace probably represents ANS activity that is prevalent most of the time; therefore, it is more useful in disease severity prediction [19,20]. In our study, we observed significant differences in anxiety scores among the various groups regarding the severity of COVID-19 based on CAS, as well as a significant correlation between the scores of CAS and HRV parameters. A direct positive correlation was observed between the CAS scores and sympathetic indices and a negative correlation between the CAS scores and parasympathetic indices. Therefore, we propose that HRV and CAS scores can be used to predict the severity of COVID-19.

HRV and inflammatory markers - biomarkers

Heart rate variability (HRV) is a noninvasive tool for the assessment of health risks, including cardiac and noncardiac diseases, and physical and psychological stress. This vicious cycle results in increased inflammation, at least during the early stages. Studies have also shown that the sympathetic nervous system plays a pivotal role in decreasing inflammation at a later stage. Our study focused on the initial phases of COVID-19 and did not include the role of SNS. In the present study, we observed a similar finding, in line with a previous study conducted by Yin C. [21]. A direct positive correlation was observed between the sympathetic indices of HRV and levels of markers of inflammation, and a negative correlation was observed between the parasympathetic indices of HRV and levels of markers of inflammation. The SDNNs were substantially decreased, indicating a reduction in HRV, in the whole course of all three groups. In all three groups, decreases in HRV occurred earlier than increases in biomarkers as listed in Table 2. SDNN was negatively associated with all inflammatory biomarkers, although the relationship with the lymphocyte cell count was weakest and reached only borderline significance. The association of lymphocyte cell count with different HRV variables should further be investigated to understand the underlying mechanisms of the relationship between lymphocytes and autonomic function.

In our study, indices of short-term HRV reflecting sympathetic activity revealed a positive correlation between neutrophil%, lymphocyte%, monocyte%, eosinophil%, NLR, serum ferritin, CRP, IL-6, LDH, procalcitonin, and MPV and a negative correlation with Hb, PCV, and MCHC. Similar findings are seen in the study conducted by Aeschbacher et al. [22].

These results support our hypothesis that higher physiological stress is related to higher neutrophil%, serum ferritin, CRP, IL-6, MPV, and lower monocyte%, lymphocyte%, and eosinophil%, LDH, Hb, PCV, MCHC, which are indicative of immune suppression. These findings suggest that HRV analysis can potentially be used to determine the presence of acute and chronic stress and related immune dysfunction [23].

HRV, a complex measure that reflects parasympathetic and sympathetic activity, is linked to inflammatory indices. Acute stress leads to stimulation of the sympathetic-adrenal-medullary axis, resulting in the release of catecholamines and other pro-inflammatory mediators, causing an increase in the heart rate and a decrease in HRV. Chronic stress leads to stimulation of the hypothalamic-pituitary-adrenal axis, resulting in the release of glucocorticoids and a decrease in lymphocyte and monocyte percentages, thus decreasing immunity and inflammation [24]. A study done by Hasty et al. consistently found a correlation between a >40% decrease in HRV and a subsequent 50% increase in CRP [25]. In addition, Aeschbacher et al. in their study concluded that inflammatory parameters are strongly associated with increased HR and decreased HRV [26].

Bourdillon et al. suggested that the strict COVID-19 lockdown likely had opposite effects on the French population, as 20% of participants improved their parasympathetic activation (RMSSD, HF) and positively rated this period, while 80% showed altered responses and deteriorated well-being [27]. The changes in HRV parameters during and after the lockdown period were consistent with the subjective well-being responses. The observed recordings may reflect a large variety of responses (anxiety, anticipatory stress, changes in physical activity, etc.), which are beyond the scope of the present study. However, these results confirmed the usefulness of HRV as a noninvasive method for monitoring the well-being and health of this population.

Limitations of the study

Limitations of the study are HRV, and inflammatory biomarkers cannot be addressed. Healthy controls (age- and sex-matched) were not used to compare the elevated community anxiety and stress during COVID-19 pandemic. Prospective studies should then be carried out to better explore the prediction effect of heart rate variability and CAS on COVID-19 severity. We encourage future studies to use heart rate variability as a tool for the prediction of the severity of inflammatory diseases.

## Conclusions

It is necessary to understand the anxiety scores and stress levels in COVID-19 patients. Psychological support should be initiated for these patients as they develop anxiety and fear of the disease. We find biomarkers that are associated with lower HRV in COVID-19. Importantly, lower HRV was independently associated with the severity of COVID-19. This study highlighted the significance of biomarkers WBC count, MPV, PWD, platelet count, NLR, serum ferritin, D-dimer, CRP, IL-6, LDH, and PCT levels in predicting the severity of COVID-19 patients. Measuring CAS, HRV, and biomarker levels in COVID-19 patients upon hospital admission has been shown to have considerable diagnostic importance. Thus, CAS and HRV provide additional valuable prognostic information that can contribute to the assessment of risks, such as hematological, biochemical, coagulation, and inflammatory biomarkers in COVID-19. This study proposes that more studies should be done to further explore the practical utility of HRV as a clinical biomarker to help identify the severity of the disease.
